# Normative Data for the Adult Turkish Population and Validation Study in Mild Cognitive Impairment and Alzheimer's Disease of the TDQ‐30 Tr, a Color Picture‐naming Test for Adults and the Elderly

**DOI:** 10.1002/brb3.70718

**Published:** 2025-08-12

**Authors:** Elif İkbal Eskioglu, Fenise Selin Karali, Samet Tosun, Nilgün Cinar, Joël Macoir

**Affiliations:** ^1^ Faculty of Health Sciences, Department of Speech and Language Therapy Biruni University İstanbul Türkiye; ^2^ Department of Speech and Language Therapy Istanbul Medipol University Graduate School of Health Sciences İstanbul Türkiye; ^3^ School of Medicine, Department of Neurology Maltepe University İstanbul Türkiye; ^4^ École Des Sciences de La Réadaptation, Faculté de Médecine Université Laval Québec QC Canada; ^5^ CERVO Brain Research Centre Québec Québec QC Canada

**Keywords:** anomia, assessment, mild anomia, naming, normative data, test validity

## Abstract

**Background:**

Individuals with mild anomia often have difficulty finding words during conversations, even when they get normal results on standard language evaluations. The high frequency and familiarity of target objects may lead to the insensitivity of some naming assessments, complicating diagnosis. This study aims to adapt the Test de dénomination de Québec‐30 images (TDQ‐30) into Turkish, develop normative data adapted to the Turkish population, and determine its validity in Turkish‐speaking patients.

**Methods:**

Data were collected from a total of 464 participants (414 healthy controls, 25 with Alzheimer's disease (AD), and 25 with mild cognitive impairment (MCI)) by using the Montreal Cognitive Assessment (MoCA), Boston Naming Test (BNT), Detection Test for Language Impairments in Adults and the Aged‐Turkish version (DTLA‐Tr), and Test de dénomination de Québec‐30‐Turkish version (TDQ‐30 Tr).

**Results:**

In Study 1, TDQ‐30 was translated into Turkish and checked for cultural appropriateness for the Turkish community. In study 2, the adapted version, TDQ‐30 tr, was applied to 414 healthy adults and elderly individuals to establish normative data for the test. In study 3, the analyses made known the validity of TDQ‐30 Tr, and it was found that TDQ‐30 can differentiate between healthy participants and those with AD and MCI, with healthy participants showing better results.

**Conclusion:**

In summary, the TDQ‐30 Tr is an effective and dependable tool for identifying mild anomia associated with neurological impairments in Turkish‐speaking adults and the elderly. Its ease of use makes it a vital instrument for improving evaluation tools in research and clinical settings in Turkey.

## Graphical abstract

This study aims to adapt the Test de dénomination de Québec‐30 images (TDQ‐30) into Turkish, develop normative data adapted to the Turkish population, and determine its validity in Turkish‐speaking patients. Data was collected from 414 healthy participants, 25 Alzheimer's disease (AD) patients and 25 mild cognitive impairment (MCI) patients by using Montreal Cognitive Assessment (MoCA), DTLA‐Tr, and TDQ‐30 Tr. Percentiles and cutoff scores were calculated by considering age, education level and sex. This study proves TDQ‐30 Tr is an effective and dependable tool for identifying mild anomia.

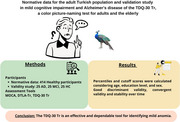

## 1 Introduction

Acquired speech and language difficulties are a significant manifestation of post‐stroke aphasia (Sheppard & Sebestian, 2021) but also of neurodegenerative disorders, including mild cognitive impairment (MCI) (Taler and Phillips, 2008), Alzheimer's Disease (AD) (Boschi et al. 2017), frontotemporal dementia (Geraudie et al. 2021), vascular dementia (Martínez‐Nicolás et al., 2022), and primary progressive aphasia (PPA) (Mesulam et al., 2014). Every neurodegenerative disorder has various symptoms that advance uniquely in each person (Gumus et al. 2024). Therefore, comprehending the manifestations of speech and language impairments in neurodegenerative disorders and their correlation with clinical symptoms might enhance the clinical comprehension and characterization of these conditions (Bertola et al. 2014; Fraser et al. 2014).

Naming difficulties, also referred to as anomia, are one of the most significant indicators of neurodegeneration (Macoir 2021). Identifying the functional origin of anomia requires recourse to models of word production that include several processing stages, namely conceptual preparation, lemma retrieval (i.e., the access and selection of an abstract lexical unit defined by semantic and syntactic information), retrieval of the phonological word‐form, phonological encoding (i.e., syllabification and segmentation), and motor speech planning and articulation (Caramazza, 1997; Dell and O'Seaghdha, 1992; Levelt et al., 1999; Roelofs, 1992). Naming difficulties in major neurodegenerative diseases may indicate a disruption at one or more of these stages. Anomia may occur after damage impacting several locations throughout the left cortical and subcortical structures (Kertesz 1979), such as the thalamus. Evidence indicates that lesions in the left temporal region are linked to abnormalities in noun retrieval (Damasio and Tranel 1993; Tranel et al. 1997), whereas lesions in the left inferior frontal area are connected with impairments in verb retrieval (Tranel et al. 1997; Caramazza and Hillis 1990; Wilshire and Coslett 2000). Numerous individuals, however, encounter deficits in the recall of both nouns and verbs.

A decline in lexical access, defined as the retrieval of words from memory, is seen in normal aging (Au et al., 1995; Burke, 1997), especially concerning the names of people (Shafto et al., 2007). Moreover, the challenge of recalling the names of individuals and things during discussion is among the most frequently expressed by the elderly (Jonker et al. 2000). This decline may be partially ascribed to a deterioration in executive control (Maril, Simons, Weaver, & Schacter, 2005), cognitive functions (Craik and Bialystok 2006), and language difficulties (Gleichgerrcht et al., 2015). However, anomia can be an indicator for neurodegenerative diseases such as PPA (Mesulam 2001), MCI (Karalı et al., 2023), or AD (Cummings and Cole 2002). It is also evident in other neurological disorders.

The severity of anomia varies among individuals according to the underlying pathology and the duration of the disease. Mild anomia is characterized by a slight impairment in the automatic retrieval of words or names. Individuals with mild anomia may have a clear idea of what they want to express but have difficulty finding the appropriate word. This phenomenon can occur in ordinary conversations and can lead to pauses or the recourse to circumlocutions (Ardila and Rosselli 1993). Individuals with mild anomia frequently experience challenges in word retrieval during conversations, despite achieving normal scores on conventional word‐finding assessments, such as the Western Aphasia Battery (Kertesz 1982) and the Boston Naming Test (Kaplan et al. 1983) (Hunting‐Pompon et al. 2011). However, these tests did not control for psycholinguistic variables known to influence word retrieval.

The high frequency and/or familiarity of the target items may contribute to the insensitivity of some naming tests, making the diagnosis of anomia more difficult. For example, difficulties in naming were seen in patients with MCI only for low‐frequency objects (Adlam, Bozeat, Arnold, Watson, & Hodges, 2006). Mild anomia is therefore often underdiagnosed, mostly because of the inadequate sensitivity of naming assessments (Moore, 2003; Hunting‐Pompon et al. 2011).

In Turkey, the Boston Naming Test (BNT), Comprehensive Aphasia Test (CAT), and Afazi Dil Değerlendirmesi (ADD) naming subtests are commonly used for language assessment in adults and older people. However, these tests often include high‐frequency and highly familiar items, which reduces their sensitivity in detecting subtle word‐finding difficulties such as mild anomia. As reported by Hunting‐Pompon et al. (2011) and Moore (2003), individuals with mild lexical retrieval difficulties may perform within normal limits on these conventional naming assessments. Similarly, Adlam et al. (2006) demonstrated that individuals with MCI showed naming impairments primarily for low‐frequency stimuli, highlighting the need for naming tasks that include psycholinguistically controlled items to reveal subtle deficits.

This study aims to adapt the Test de dénomination de Québec‐30 images (Quebec naming test‐30 pictures, TDQ‐30) (Macoir et al. 2021) into Turkish, to develop normative data adapted to the Turkish population, and to determine its validity in Turkish‐speaking patients. The TDQ‐30 was specifically designed to detect mild word‐finding deficits in adults and elderly people by using low‐frequency and low‐familiarity items. Since its publication, the test has been used in various clinical and non‐clinical populations (e.g., Brisebois et al. 2023; Mulet‐Perreault et al. 2025). This study received ethical clearance from the Ethics Committee of XXX University (Protocol Number: 2015‐KAEK‐80‐23‐26).

### 1.1 Study 1: Turkish Adaptation of the TDQ‐30

Initially, we translated all the TDQ‐30 items into Turkish and then evaluated their cultural appropriateness and frequency in Turkish. To assess the face validity, we asked five experienced speech and language pathologists (SLP) to complete a questionnaire on the name and cultural appropriateness of the test items. This questionnaire helped to create the final version of the TDQ‐30 by changing six items for which the superordinate semantic category was retained. Table 1 displays the changes made to the original version of the TDQ‐30 Tr. These changes were related to 6 specific items. The words “arachide” (peanut), “extincteur” (fire extinguisher), “glacière” (icebox), “huitre” (oyster), “arrosoir” (watering can), “hameçon” (fish hook) were replaced with “artichoke,” “headpin,” “hose,” “lizard,” “barrel,” and “anchor” respectively. As in the original version of the test, the photos of these 6 specific items were retrieved from the Bank of Standardized Stimuli (BOSS), established by Brodeur and colleagues (2010, 2014).

**TABLE 1:** Item list of the original and Turkish version of the TDQ‐30.

Item number	Original version in French	English version	Turkish version		Final Turkish version	Semantic category
1	arachide	Peanut	yer fıstığı	Changed to	enginar [artichoke]	Man‐made
2	cactus	Cactus	kaktüs		kaktüs	Biological
3	cerf‐volant	Kite	uçurtma		uçurtma	Man‐made
4	chauve‐souris	Bat	yarasa		yarasa	Biological
5	extincteur	Fire extinguisher	yangın söndürücü	Changed to	labut [headpin]	Biological
6	pélican	Pelican	pelikan		pelikan	Man‐made
7	trèfle	Clover	yonca		yonca	Man‐made
8	harmonica	Harmonica	armonika		armonika	Biological
9	glacière	Icebox	soğuk tutucu	Changed to	hortum [hose]	Man‐made
10	chandelier	Candlestick	şamdan		şamdan	Man‐made
11	gland	Acorn	palamut		palamut	Man‐made
12	seringue	Syringe	şırınga		şırınga	Man‐made
13	poireau	Leek	pırasa		pırasa	Biological
14	fourche	Pitchfork	tırmık		tırmık	Man‐made
15	huitre	Oyster	istiridye	Changed to	kertenkele [lizard]	Man‐made
16	perceuse	Drill	matkap		matkap	Biological
17	libellule	Dragonfly	yusufçuk		yusufçuk	Biological
18	accordéon	Accordion	akordiyon		akordiyon	Biological
19	navet	Turnip	turp		turp	Man‐made
20	voilier	Sailboat	yelkenli		yelkenli	Biological
21	panda	Panda	panda		panda	Biological
22	arrosoir	Watering can	sulama kabı	Changed to	fıçı [barrel]	Man‐made
23	paon	Peacock	tavuskuşu		tavuskuşu	Biological
24	hameçon	Fish hook	olta iğnesi	Changed to	çıpa [anchor]	Man‐made
25	poivron	Pepper	biber		biber	Biological
26	cintre	Hanger	askı		askı	Biological
27	fougère	Fern	eğreltiotu		eğreltiotu	Man‐made
28	trampoline	Trampoline	trambolin		trambolin	Man‐made
29	ventilateur	Fan	vantilatör		vantilatör	Man‐made
30	autruche	Ostrich	devekuşu		devekuşu	Man‐made

### 1.2 Study 2: Normative Data of the Turkish TDQ‐30

The aim of Study 2 was to develop normative data for the TDQ‐30 Tr adapted to adult and aged populations from Turkey.

## 2 Method

The initial study sample included 423 Turkish‐speaking adults aged 18 years and older, all of whom lived in the community and were in good health. To qualify, participants had to (1) be assessed as healthy by a neurologist, (2) give informed consent, (3) have sufficient sensory abilities to perform the tasks, (4) be native Turkish speakers, and (5) not have suffered head trauma in the past two years. Participants were excluded if their Montreal Cognitive Assessment (MoCA) was below the thresholds set by Kaya et al. (2014) based on the level of education in Turkey: Primary School = 17/30; Secondary/High School = 20/30; university = 22/30. In addition, those whose scores on the Detection Test for Language Impairments in Adults and the Aged (DTLA) were below the thresholds set by Karalı et al. (2024), which are also based on the Turkish education system, were excluded: Elementary school = 76/100; secondary/high school = 82/100; University = 80/100. All participants reported being mentally and physically healthy, with no neurological disorders, untreated psychiatric illnesses, traumatic brain injuries, or unresolved medical problems that could impair cognitive performance.

### 2.1 Material and Procedure

This study is part of a larger validation and normative project that encompasses various language assessments. Participants participated in two assessment sessions of 60 min. First, they gave written informed consent and completed a sociodemographic questionnaire. Then the MoCA and DTLA were administered to assess cognitive and language health. Participants were then assessed with the TDQ‐60 (Karalı et al. 2025; Macoir et al., 2018), the Turkish version of the Boston Naming Test ‐ 30 items, the BECLA‐TR, and the TDQ‐60. The BNT was used to determine the convergent validity of the TDQ‐30 TR (see Study 3). Each participant underwent testing in a single session in a quiet environment, either at home or in a research facility. The TDQ‐30 Tr was conducted using a PowerPoint presentation or colorful paper booklet, while the BNT was conducted using a paper booklet. Participants were not interrupted during the tasks, and instructions were repeated as needed. They were asked to name each picture in the two tests with a single noun after it was shown. Responses were carefully recorded verbatim.

Unlike the Boston Naming Test (BNT), the TDQ‐30 Tr is administered without any semantic or phonological cues. Participants are asked to name each picture spontaneously upon presentation, with no additional prompts or hints. This administration format aims to preserve the test's sensitivity to mild word‐finding difficulties.

### 2.2 Statistical Analysis

The primary variable for normative analysis was the total score on the TDQ‐30 Tr. Data distributions were first examined for skewness using the Shapiro–Wilk test and visualized with histograms. Although the TDQ‐30 Tr scores were moderately skewed, we chose not to apply transformations, as regression‐based normative approaches are robust to non‐normality of the dependent variable, provided residuals are approximately normal. This was confirmed through visual inspection (histograms and Q‐Q plots) and supported the use of linear regression.

To develop regression‐based normative data for the TDQ‐30, a multiple linear regression analysis was conducted using centered values of age and years of education as continuous predictors of total test performance. Gender was initially included in the model but did not contribute significantly and was therefore excluded from the final model. Centering was applied to reduce multicollinearity and improve interpretability of the intercept. The analysis was performed using Python (statsmodels package) on the full normative dataset comprising 414 healthy Turkish‐speaking adults. The model included no interaction term and did not apply standardization to the predictors. This approach allows for the individual adjustment of performance based on continuous sociodemographic variables.

In addition, to accommodate clinical users less familiar with regression‐based methods, normative percentiles were calculated using Jamovi 2.5 (The Jamovi Project 2024), with the significance level set at *α* = 0.05. Percentiles were derived by stratifying the sample into nine demographic subgroups based on three levels of education (≤ 10 years, 11–13 years, and ≥ 14 years) and three age groups (18–40, 41–60, and 61–81 years). This 3×3 classification was selected to balance clinical interpretability with statistical reliability. The three‐tiered education model captures meaningful variation in formal schooling across the adult Turkish population and is consistent with standard practices in neuropsychological norming. Similarly, the three age bands were defined to ensure adequate sample sizes within each subgroup while minimizing within‐cell variability in percentile estimation. The resulting stratification provided robust normative reference values suitable for clinical use. The eighth percentile was adopted as the cutoff for identifying below‐normal performance, in line with recent recommendations for interpreting neuropsychological test scores (Guilmette et al., 2020).

## 3 Results

Nine participants were excluded from the initial sample due to subthreshold scores on the MoCA (*n* = 4) and/or the DTLA‐Tr (*n* = 5), yielding a final normative sample of 414 participants (250 women, 164 men), aged from 18 to 81 years (M = 46.3, SD = 19.2), and from 8 to 21 years of education (M = 11.9, SD = 3.1). Table 2 shows that the participants were well represented in the different age groups and educational levels.

**TABLE 2:** Distribution of participants in the normative sample.

Characteristics	*n* (%)
**Age (years)** 18–32 33–48 49–64 65–81	124 (29.95) 94 (22.70) 107 (25.84) 89 (21.50)
**Women**	250 (60.40)
**Education (years)** Primary and middle school (8 years of education) High school (9–14 years of education) University (15 years of education)	121 (29.23) 147 (35.51) 146 (35.27)
**Cognitive screening** MoCA score DTLA‐Tr score	**Mean (SD)** 25.80 (3.42) [Min = 17; Max = 30] 96.60 (4.81) [Min = 76; Max = 100]
**TDQ‐60 Tr Total score**	56.6 (3.55) [Min = 32; Max = 60]

Visual inspection of residuals from the regression model revealed an approximately normal distribution. The Shapiro–Wilk test was statistically significant (W = 0.982, *p* < 0.001), which was expected given the large sample size. No problematic skewness or outliers were observed in histograms or Q‐Q plots. The final regression model included age and education as predictors of TDQ‐30 score. Age was negatively associated with performance (*p* = 0.053), and education had a strong positive effect (*p* < 0.001). Although the effect of age was marginally significant, it was retained in the model due to its theoretical relevance and consistent negative association with lexical retrieval performance across the adult lifespan, as reported in prior research (e.g., Albert et al. 1988; Tsang and Lee 2003). Including age in the model ensures more accurate individual predictions, particularly in clinical contexts where even subtle age‐related effects may impact interpretation.

The model allowed for the calculation of predicted scores, standardized residuals (i.e., Z‐scores), and corrected percentiles. This equation enables clinicians to interpret a participant's performance relative to the expected value based on their age and education. To facilitate the calculation of Z‐scores based on the regression formula, a Microsoft Excel spreadsheet with automatic formulas was created. This file can be requested from the corresponding author of this article.

Table 3 presents the regression coefficients, and Table 4 provides the Z‐score equation.

**TABLE 3:** Regression coefficients for the TDQ‐30 Tr.

	*B*	*t*	*p*	95% CI
Age	−0.025	−1.94	= 0.053	[‐0.051, 0.000]
Education	0.500	6.25	< 0.001	[+0.343, + 0.657]

Intercept = 22.203; Square root of the mean square residual = 4.94.

**TABLE 4:** Comparison of HC, MCI and AD participants on sociodemographic variables and TDQ‐30 Tr.

Characteristics	HC (*n*=25)	MCI (*n*=25)	AD (*n*=25)	*p*	Effect size
Age, mean (SD)	70.6 (5.16)	70.4 (6.84)	71.1 (5.11)	0.867	ε^2^ = 0.0038
Education, mean (SD)	10.8 (2.93)	12.8 (2.57)	10.6 (2.83)	0.014	ε^2^ = 0.116
Female, *n* (%)	17 (68.00)	10 (40.00)	15 (60.00)	0.12	
MoCA, mean (SD)	24.1 (3.35)	18.8 (1.74)	13.9 (2.96)	< 0.001^a^, ^b^, ^c^	*n_p_^2^* = 0.702
DTLA‐Tr, mean (SD)	95.1 (5.16)	89.2 (7.17)	76.7 (14.40)	< 0.001^a^, ^b^, ^c^	ε^2^ = 0.42
TDQ‐30 Tr Total score (30) Natural concepts (15) Man‐made concepts (15)	21.8 (4.67) 10.5 (2.77) 11.2 (2.45)	17.1 (4.90) 8.04 (2.65) 9.04 (2.70)	14.7 (4.09) 6.88 (2.39) 7.84 (2.34)	<.001^a^, ^b^ <.001^a^, ^b^ <.001^a^, ^b^	*n_p_^2^* = .300 *n_p_^2^* = .261 *n_p_^2^* = .248

**Abbreviations**: AD = Alzheimer's Disease, HC = healthy controls, MCI = mild cognitive impairment,

^a^ Symbols for significant post hoc group differences: HC versus MCI.

^b^ Symbols for significant post hoc group differences: HC versus AD.

^c^ Symbols for significant post hoc group differences: MCI versus AD.

To illustrate how the regression‐based normative model distinguishes between normal and impaired performance, two fictive cases involving 65‐year‐old men with 8 years of education were analyzed. Although both share the same demographic characteristics, their raw TDQ‐30 scores differ, leading to contrasting interpretations. In the first case, the individual obtained a TDQ‐30 score of 17. Based on the model, the predicted score for someone of his age and education level is 19.81, resulting in a Z‐score of ‐0.57 and a percentile rank of approximately the twenty‐nineth percentile. This performance, though below average, remains within the normal range. In contrast, the second individual obtained a substantially lower score of 12. With the same predicted score of 19.81, his Z‐score was ‐1.58, corresponding to the sixth percentile. This places his performance below the eighth percentile cutoff and supports an interpretation of impaired lexical‐semantic access relative to age‐ and education‐adjusted expectations.

In parallel, percentile‐based normative tables were produced for 9 demographic strata defined by age and education. These tables (see Table 5) provide an accessible alternative to regression models and can support clinical decision‐making when computational tools are not available.

**TABLE 5:** Descriptive statistics (mean and standard deviation) of the participants for the normative study as a function of age and educational level.

Age/education group		18–40/ 11–13 years (*n*=56)			41–60/ 11–13 years (*n*=31)			61–81 / 11–13 years (*n*=54)	
Mean score (SD)	20.42 (5.24)	23.05 (4.54)	24.03 (4.54)	19.85 (5.48)	23.26 (5.4)	25.13 (4.22)	18.85 (5.39)	21.33 (5.15)	22.91 (4.79)
Percentiles									
First	10	13	14	11	9	15	9	10	11
Second	11	15	14	12	10	16	9	11	12
Fifth	11	16	16	12	14	17	10	13	15
**Eighth (cutoff)**	**11**	**17**	**17**	**13**	**16**	**18**	**11**	**15**	**16**
Tenth	13	17	17	14	17	19	12	15	16
Fifteenth	15	18	19	14	18	20	14	16	19
Twenty‐fifth	18	20	21	15	20	23	15	17	20
Thirty‐fifth	19	21	22	16	22	25	17	20	21
Forty‐fifth	20	23	24	20	25	26	18	21	23
Fiftieth	20	23	25	20	25	27	18	22	23
Fifty‐fifth	21	24	25	20	26	27	20	22	24
Sixty‐fifth	23	25	26	22	26	27	21	23	26
Seventy‐fifth	24	27	28	25	27	28	23	25	27
Eighty‐fifth	27	28	29	26	28	29	24	27	28
Ninetieth	27	29	30	27	29	30	25	28	29
Ninety‐fifth	28	30	30	29	29	30	27	29	29
Ninety‐eighth	28	30	30	29	29	30	30	30	30
Ninety‐ninth	29	30	30	29	30	30	30	30	30

*Note*: In cases where the score at the eighth percentile is identical to that of a higher percentile (e.g., the tenth or fifth), the next lower percentile with a distinct score should be used to determine below‐normal performance. This approach avoids overestimating impairment when percentile scores are tied due to score discreteness.

**Abbreviations**: Cutoff = suggested cutoff score calculated as the eighth percentile (score equal to or below it is under normal performance limits),

SD = standard deviation,

### 3.1 Study 3: Validity of the TDQ‐30 TR

The objective of Study 3 was to determine the known‐group discriminant validity, convergent validity, and test‐retest validity of the TDQ‐30 Tr within the Turkish population.

## 4 Methods

### 4.1 Known‐group Discriminant Validity

To determine known‐group discriminant validity, we analyzed whether TDQ‐30 Tr scores differed between healthy controls (HCs), individuals with MCI, and those with AD. Since semantic processing and lexical access are impaired in these conditions (Taller and Phillips 2008), we hypothesized that participants with MCI and AD would score significantly lower on the TDQ‐30 Tr compared to HCs. Table 4 provides a detailed overview of the sociodemographic characteristics of the participants across the three groups. The same three groups of participants were recruited in an earlier study on the validity of the TDQ‐60 Tr, another picture naming test. (Karalı et al., 2025).

#### 4.1.1 Participants

The MCI group consisted of 25 participants who had been diagnosed on the basis of the clinical criteria of Winblad et al. (2004). These criteria included (1) cognitive concerns indicating a change in cognition reported by the patient, an informant, or the clinician (evidence of decline over time); (2) objective evidence of impairment (more than ‐1.5 standard deviation [SD] based on age, sex, and education norms) in one or more cognitive domains, including episodic memory; (3) no significant impairment in functional abilities as per clinical consensus and the Alzheimer's Disease Cooperative Study‐Activities of Daily Living (ADCS‐ADL); and (4) not meeting the criteria for dementia. Although many individuals in the sample showed memory impairments, this was not a mandatory inclusion criterion, and the group may have included both amnestic and non‐amnestic MCI presentations.

The AD group consisted of 25 individuals diagnosed with probable AD, based on current diagnostic criteria (McKhann et al. 2011). This diagnosis was confirmed through patients' medical records and history, including a medical doctor's diagnosis and/or receiving approved pharmacological dementia treatment. All participants were at the mild stage of the AD. All participants were at the mild stage of AD, as determined by the clinical judgment of the neurologist in our research team, in accordance with McKhann et al. (2011) criteria, and supported by MoCA scores between 18 and 25, as well as functional performance consistent with AD mild cognitive decline.

The HC group consisted of 25 participants from Study 2 (normative data), selected to match the AD and MCI participants in terms of demographic characteristics, preventing bias. They were in good physical and mental health, reported no significant subjective cognitive complaints, and showed normal cognitive performance on the MoCA test, based on Turkish normative data.

The exclusion criteria for all participants were: (1) a history of moderate or severe traumatic brain injury, (2) a history of clinically diagnosed cerebrovascular disease, including stroke, with suspected impact on cognition, (3) a history of delirium within the past 6 months, (4) a history of intracranial surgery, (5) a history of neurological disorders of cerebral origin not investigated in the study, (6) a history of encephalitis or bacterial meningitis, (7) unstable metabolic or medical conditions (e.g., untreated hypothyroidism or diabetes), (8) an active and unstable psychiatric syndrome, (9) alcoholism or substance abuse, (10) uncorrected vision or hearing impairments, (11) a cancer treatment in the past 12 months; (12) a general anesthesia in the past 6 months; (13) a history of psychotic symptoms or manic episodes and 14) inability of the participant to provide consent due to inaptitude.

#### 4.1.2 Procedure

The participants in the three groups were administered three tests in the same order, namely the MoCA, the DTLA, and finally the TDQ‐30 Tr.

#### 4.1.3 Statistical Analyses

Differences in sex distribution across the three groups were assessed using chi‐square analysis. Kruskal–Wallis tests compared the groups based on age, educational level, and DTLA score. Analysis of variance (ANOVA) was employed to compare MoCA, DTLA and TDQ‐30 Tr performances (total score and scores for natural and man‐made concepts). Pairwise comparisons utilized Dwass–Steel–Critchlow–Fligner post hoc tests or paired *t*‐tests with Bonferroni correction. Effect sizes were reported as partial eta squared (η^2^) and interpreted per Cohen (1988): 0.01 to 0.06 indicates a small effect, 0.06 to 0.14 a moderate effect, and above 0.14 a strong effect. All statistical analyses were performed using Jamovi 2.5. (The Jamovi Project 2024), with an alpha level of 0.05.

## 5 Results

Table 4 demonstrates that the three groups were statistically equivalent in terms of sex (*χ^2^* = 4.21, *df* = 2, *p* = 0.12) and age (*χ^2^* = 0.285, *df* = 2, *p* = 0.87). However, there was a significant difference in educational level (*χ^2^* = 8.58, *df* = 2, *p* = 0.014). The Dwass–Steel–Critchlow–Fligner post hoc tests revealed that the age of AD participants was comparable to HCs (*W* = 0.37, *p* = 0.96), but they were significantly older than the MCI participants (*W* = 3.75, *p* = 0.022). Additionally, the HCs were statistically older than the MCI participants (*W* = 3.39, *p* = 0.044).

As anticipated, the three groups showed significant differences in general cognition measured by the MoCA, *F*(2, 72) = 84.7, *p* < 0.001. Post‐hoc tests revealed: (a) HCs performed significantly better than participants with MCI (*p* < 0.001) and participants with AD (*p* < 0.001); (b) participants with MCI performed significantly better than those with AD (*p* < 0.01). Similarly, the groups also displayed significant differences in general language ability measured by the DTLA, *F*(2, 72) = 23.3, *p* < 0.001. Post‐hoc tests showed that participants with AD scored significantly lower than both HCs (*p* < 0.01) and participants with MCI (*p* < 0.01), while the performance of HCs and participants with MCI was statistically equivalent (*p* < 0.105).

Regarding the TDQ‐30 Tr, ANOVA indicated a significant main effect of group for the total score, *F*(2, 72) = 15.4, *p* < 0.001. Post‐hoc tests revealed that HCs performed significantly better than both participants with MCI (*p* = 0.002) and those with AD (*p* < 0.001). However, the performance of participants with MCI was comparable to those with AD (*p* = 0.215). The results by semantic category also revealed main effects of the group for the score on natural concepts, *F* (2, 72) = 12.7, *p* < 0.001, and on man‐made concepts, *F*(2, 72) = 11.9, *p* < 0.001. For natural concepts, the post‐hoc tests showed (a) HCs performed significantly better than participants with MCI (*p* = 0.004) and participants with AD (*p* < 0.001); (b) the performance of participants with MCI was statistically similar to that of participants with AD (*p* = 0.361). For man‐made concepts, the post‐hoc tests indicated (a) HCs outperformed participants with AD (*p* < 0.001) and participants with MCI (*p* = 0.008); (b) the performance of participants with MCI was statistically comparable to that of participants with AD (*p* = 0.283).

### 5.1 Convergent Validity

Convergent validity was established by administering two picture naming tests, the TDQ‐30 Tr and the Turkish version of the Boston Naming Test (Soylu and Cangöz 2018). Given that both tests require similar cognitive processes, such as visual recognition, semantic activation, and lexical access, a positive correlation was anticipated.

#### 5.1.1 Participants

Both naming tests were administered to the 414 healthy participants in the normative study (see Study 2) as well as to the 25 participants with MCI and the 25 participants with AD in the known‐group discriminant validity study.

#### 5.1.2 Statistical Analyses

As the data in the two tests were normally distributed, the relationship between the participants' performance in the two tests was analyzed using the Pearson product‐moment correlation.

#### 5.1.3 Results

As expected, the TDQ‐30 Tr correlated significantly and positively (Pearson *r* = 0.840, *p* < 0.001) with the BNT‐30, indicating that the two tests measure the same cognitive construct.

### 5.2 Predictive and Clinical Utility of the TDQ‐30 Tr

To further establish the clinical utility of the TDQ‐30 Tr, we examined its ability to predict naming impairment, using the BNT‐30 as an external criterion. While convergent validity was supported by strong correlations between the two measures, predictive validity analyses were conducted to determine whether TDQ‐30 Tr scores could accurately classify individuals with anomia. This approach provides a more clinically meaningful evaluation of the TDQ‐30 Tr's diagnostic relevance, particularly in identifying individuals at risk for language impairment across both healthy and clinical populations.

#### 5.2.1 Participants

The analyses were conducted on the data from the 414 healthy participants included in the normative study (see Study 2), as well as the 25 participants with MCI and the 25 participants with AD from the known‐group discriminant validity study.

#### 5.2.2 Statistical Analyses

A binary logistic regression was conducted. The model examined whether TDQ‐30 Tr total scores predicted the presence of anomia, defined as a score of 25 or below on the BNT‐30, a commonly used clinical cutoff (Mack et al. 1992; Saxton et al. 2000). To further evaluate the diagnostic accuracy of the TDQ‐30 Tr, we calculated sensitivity, specificity, overall classification accuracy, and the area under the receiver operating characteristic curve (AUC) based on predicted probabilities from the logistic model. These analyses were repeated using two stricter, data‐driven thresholds for defining anomia based on the distribution of BNT scores in the healthy control group: a score ≤ 19 (1.5 standard deviations below the mean) and ≤ 18 (fifth percentile). All analyses were performed using Python 3.10 with an alpha level of 0.05.

#### 5.2.3 Results

Binary logistic regression analyses revealed that TDQ‐30 Tr total scores significantly predicted naming impairment across all three BNT‐based anomia definitions. When using the conventional cutoff of ≤ 25 on the BNT‐30, the TDQ‐30 Tr was a strong and significant predictor of anomia (*b* = ‐0.447, *p* < 0.001), with a pseudo *R^2^* of 0.44, indicating a robust model fit. Diagnostic accuracy analyses yielded a sensitivity of 84.3%, a specificity of 87.8%, an overall accuracy of 87.3%, and an area under the curve (AUC) of 0.92, suggesting excellent discriminative power.

When applying the more conservative, data‐driven thresholds of ≤ 19 and ≤ 18 on the BNT‐30, the TDQ‐30 Tr remained a significant predictor of anomia (*b* = ‐0.462 and ‐0.437, respectively; both *p* < 0.001). These models demonstrated increased specificity (92.6% and 91.5%) and overall accuracy (93.3% and 91.8%), with slightly reduced sensitivity (72.7% and 71.1%). AUC values remained high for both models (0.91 and 0.89), indicating strong predictive validity.

These results confirm that the TDQ‐30 Tr reliably identifies individuals with naming impairment and may serve as an efficient screening tool in both research and clinical settings.

### 5.3 Test‐retest Reliability

Test‐retest reliability refers to the consistency of scores over time and was assessed by comparing TDQ‐30 Tr performance across two administrations conducted at an interval of 2 weeks to 1 month.

#### 5.3.1 Participants

This procedure was performed on a group of 32 healthy participants (7 men, 25 women) with an average age of 38.1 years (SD = 16.1), an average educational level of 13.0 years (SD = 2.93), an average MoCA score of 27.2 (SD = 3.05), and an average DTLA score of 97.2 (SD = 5.17). These participants, who also participated in the normative study (see Study 2), agreed to be tested twice with the same test.

#### 5.3.2 Statistical Analyses

Test‐retest reliability was assessed by comparing TDQ‐30 scores at Time 1 (T1) and Time 2 (T2) using both Bland–Altman analysis [39] and the intraclass correlation coefficient (ICC). The Bland–Altman method was used to assess agreement between TDQ‐30 scores at Time 1 (T1) and Time 2 (T2) by plotting the mean of each score pair against their difference. This analysis allowed for the visual detection of systematic bias, evaluation of the distribution of score differences around zero, and estimation of the 95% limits of agreement (mean difference ± 1.96 × SD), representing the expected range of variation between test and retest scores. The ICC quantified the consistency of scores over time.

#### 5.3.3 Results

Bland–Altman analysis showed that the 95% limits of agreement for the TDQ‐30 Tr ranged from ‐6.53 [95% CI ‐8.43, ‐4.62] to 5.43 [95% CI 3.56, 7.54], with all data points falling within this range. The mean difference (bias) between T1 and T2 scores was ‐0.53 [95% CI ‐1.63, 0.57], and the distribution of differences around the mean was relatively symmetrical, suggesting no systematic bias and good agreement between test and retest administrations. There was no significant difference in the performance of healthy participants between T1 and T2 (T1 mean = 24.8, SD = 4.60; T2 mean = 25.3, SD = 4.38; ICC = 0.768, *p* < 0.001), indicating good test‐retest reliability of the TDQ‐30 Tr over time.

## 6 Discussion

Anomia is a frequent sign of acquired aphasia and may also be a characteristic of neurodegenerative disorders, such as AD or MCI (Laine & Martin, 2006). Word retrieval is a complex process that requires access to a range of linguistic information, including semantics and phonology, to choose and construct a word (Levelt, 1999).

This research was to adapt the TDQ‐30 into Turkish, develop normative data for the Turkish adults and older people, and establish its reliability and validity. For Study 1, we translated the test items and replaced those that were not culturally appropriate for the Turkish community. During the adaptation process, six items were modified for the purpose of cultural appropriateness. The normative data for the TDQ‐30 Tr provided in Study 2 were based on the performance of a sample of 414 healthy, Turkish‐speaking adults and elderly individuals residing in the community. The sample was representative of a wide range of ages and educational levels. Percentiles and cutoff scores for the total number of responses to the TDQ‐30 Tr were calculated considering age, education level, and sex. The results of Study 3 demonstrated that the TDQ‐30 Tr has the ability to differentiate between the performance of healthy participants and participants with AD and MCI. HCs had much better results compared to both those with MCI and those with AD. In a cross‐group comparison of the superordinate semantic category (i.e., natural and man‐made concepts), the HCs performed better than the AD and MCI participants. However, the performance of the latter two groups did not differ, suggesting that the test is very sensitive to anomia in neurodegenerative diseases, even in the early stages of the disease. Finally, results of Study 3 also showed that TDQ‐30 Tr has good convergent validity with the Turkish version of the BNT and good stability over time (test‐retest reliability).

Many tests have been developed in languages that are different from our own and in countries that are culturally different from the people we want to assess. Therefore, if these tests are considered useful, they must be linguistically and culturally adapted before being used in new contexts (Ortiz‐Gutiérrez and Cruz‐Avelar 2018). Psychometrics is related to the development and validation of measurement instruments and the assessment of their reliability and validity as effective measurement tools (Ginty 2013). Consequently, consideration of psychometric characteristics is essential when assessing a person's abilities. Moreover, the assessment of language proficiency necessitates careful consideration of cultural factors, especially when assessing semantic and lexical knowledge, such as in naming tests. Cultural variations influence perceptual processing in confrontation naming (Goh and Park 2009). The familiarity of concepts can differ considerably between cultures (Lin et al. 1990), as exemplified by the contrast between Canada, the country of origin of the TDQ‐30, and Turkey.

MCI generally shows mild to moderate lexical access difficulties when speaking spontaneously and naming pictures (Convit et al. 2000). However, in certain individuals with MCI, language may remain intact or be only slightly impaired (Lambon et al., 2003). The challenge in detecting anomia in this context may be due in part to the limited sensitivity of naming tests, which often include items of high frequency and/or familiarity. However, detecting anomia in cases with very mild anomia is important, as this could lead to SLP services being offered at an early stage of the disease, thus limiting the impact on the person's autonomy and quality of life. In this regard, the adaptation and normalization of the TDQ‐Tr will prompt SLPs in Turkey to offer therapeutic treatments to patients with very mild anomia.

With the help of this new assessment tool in Turkish, future studies can be conducted to detect anomia in clinical populations known to have mild impairment in the early stages, such as multiple sclerosis and Parkinson's disease (Kristensson et al. 2024), vascular dementia (Macoir 2024), dementia with Lewy bodies (Macoir 2022), and PPA, particularly in its non‐fluent/agrammatic and logopenic variants, where anomia can be difficult to objectify (Stockbridge et al. 2023).

Although an incidental sampling method was employed, the normative data in this study are derived from a relatively large and demographically diverse sample of adults and older adults living in Turkey. The sample is broadly representative and well‐balanced in terms of age, gender, and educational background. Consequently, the findings of this study enhance our confidence in the clinical validity of our normative data. However, it's important to acknowledge that this recruitment approach may have introduced sampling bias, potentially limiting the generalizability of the results to the broader Turkish population. Additionally, although the regression‐based approach offers several advantages for generating normative data, it relies on key statistical assumptions—such as linearity, homoscedasticity, and normally distributed residuals—which, while reasonably met in our model, may still limit the accuracy of predictions in specific demographic subgroups or clinical populations. Moreover, future studies are needed to assess the diagnostic accuracy and predictive validity of the TDQ‐30 in relation to established external naming measures in order to further support its clinical utility for detecting anomia.

An additional limitation concerns the relatively small sample size in each diagnostic group in Study 3 (known‐group discriminant validity). Although the groups were balanced and enabled initial comparisons, such small samples reduce statistical power, increase the margin of error, and may limit the generalizability of the findings. Consequently, the observed group differences and conclusions regarding the TDQ‐30's ability to discriminate among healthy individuals, individuals with MCI, and those with AD should be interpreted with caution. These results should be considered preliminary evidence of discriminant validity, pending replication in larger and more diverse clinical samples.

In conclusion, the TDQ‐30 Tr is a practical and reliable instrument for diagnosing mild anomia linked to neurological deficiencies in Turkish‐speaking adults and the elderly. Its brevity and ease of administration make it a valuable addition to language assessment tools in both research and clinical settings in Turkey. Supporting Information.

## Author Contributions

All authors equally contributed to the study conception and design.

## Ethics Statement

The unpublished data reported in this manuscript were approved by the Ethics Committee of Biruni University. All procedures complied with the ethical standards established by the institution's ethics committee and the Helsinki Declaration of 1964. We obtained written informed consent from patients and their caregivers.

## Consent

All patients and their caregivers signed written informed consent for the study.

## Conflicts of Interest

The authors declare no conflicts of interest.

## Peer Review

The peer review history for this article is available at https://publons.com/publon/10.1002/brb3.70718.

## Supporting information




**Supporting Material**: brb370738‐sup‐0001‐SuppMat.xlsx

## Data Availability

The data that support the findings of this study are available from the corresponding author upon reasonable request.
